# The impact of chemotherapy dose intensity and supportive care on the risk of febrile neutropenia in patients with early stage breast cancer: a prospective cohort study

**DOI:** 10.1186/s40064-015-1165-6

**Published:** 2015-08-06

**Authors:** Eva Culakova, Marek S Poniewierski, Debra A Wolff, David C Dale, Jeffrey Crawford, Gary H Lyman

**Affiliations:** Fred Hutchinson Cancer Research Center, Hutchinson Institute for Cancer Outcomes Research, 1100 Fairview Avenue North, M3-B232, PO Box 19024, Seattle, WA 98109-1024 USA; Empire State College, Saratoga Springs, NY USA; University of Washington, Seattle, WA USA; Duke University, Durham, NC USA

**Keywords:** Chemotherapy, Toxicity neutropenia, Infection

## Abstract

**Background:**

Febrile neutropenia (FN) is a major dose-limiting toxicity of cancer chemotherapy resulting in considerable morbidity, mortality, and cost. This study evaluated the time course of neutropenic events and patterns of supportive care interventions in patients receiving chemotherapy for early-stage breast cancer treated in oncology community practices.

**Methods:**

A prospective cohort study of adult cancer patients initiating a new chemotherapy regimen was conducted at 115 US sites. Toxicity associated with chemotherapy including neutropenic and infectious complications was recorded over four cycles. Clinical interventions were recorded including reductions in chemotherapy dose intensity and use of supportive care measures.

**Results:**

A total of 1,202 patients with stage I–III breast cancer were evaluated. The majority of neutropenic (116 of 196) and infection events (179 of 325) occurred in the initial cycle. A decrease in occurrence of FN and infection was observed in the subsequent cycles, along with an increase in utilization of colony stimulating factors (CSFs), antibiotics and reductions in chemotherapy dose intensity. The overall risk of FN in all patients was 16.3%. In patients who started treatment at or near full dose intensity, the FN risk reached 21.0% without primary CSF prophylaxis and it was 9.0% with prophylaxis. There was no significant difference in FN rates by menopausal or hormone receptors status.

**Conclusions:**

The risk of neutropenic complications is greatest in the initial cycle when most patients receive full-dose chemotherapy. A decrease in neutropenic events during subsequent cycles is associated with reduced dose intensity or increased use of supportive care measures. However, the cumulative risk of FN remains high in patients with early-stage breast cancer receiving full dose chemotherapy without prophylactic measures.

## Background

Among women, breast cancer is the most prevalent cancer type with over a quarter of a million newly diagnosed patients annually and accounting for approximately 40,000 deaths each year in the US, only exceeded by lung cancer in terms of cancer-related mortality (American Cancer Society 2015; Gradishar et al. 2015). Largely attributable to advancements in diagnostic techniques and preventive care, the disease is often detected early before the development of evident distant metastases (Onitilo et al. 2013). Early-stage breast cancer (ESBC) is considered a curable malignancy. However, it is a heterogeneous disease with prognosis and also treatment decisions depending on the clinical parameters, molecular type, as well as patient’s functional status (Goldhirsch et al. 2013; Perou and Borresen-Dale 2011).

An important part of the treatment decision in ESBC is whether chemotherapy will be beneficial and if so, the choice of chemotherapy regimen (Theriault et al. 2013; Bonadonna et al. 2005; Aebi et al. 2014). All regimens before being implemented into the routine clinic practice were developed in a series of clinical trials. Deviations from the established schedule either by implementing drug dose reductions or treatment delays, resulting in reduced relative dose intensity (RDI), may compromise treatment outcome (Bonadonna et al. 1995; Budman et al. 1998; Lyman 2009). When delivered RDI falls below a certain threshold the patient may get little or no clinical benefit of treatment while still facing multiple burdens from myelotoxic treatment (Bonadonna et al. 1995).

Chemotherapy-related toxicities often cause modifications or interruption of treatment (Lyman et al. 2003, 2013a). Among the toxicities, chemotherapy-induced neutropenia, especially febrile neutropenia (FN), is considered to be a major adverse event frequently requiring hospitalization (Weycker et al. 2014; Kreys et al. 2014). It has not only negative effects on treatment schedule but is often connected with considerable morbidity, high risk of infection-related early mortality, and an increased treatment cost (Hendricks et al. 2011; Klastersky and Paesmans 2011; Kuderer et al. 2006; Lee et al. 2015; Pathak et al. 2015). Patients have the highest risk of neutropenic events at the beginning of treatment, with 50–75% of initial events happening in the first cycle of chemotherapy (Crawford et al. 2008; Vogel et al. 2005; Timmer-Bonte and Tjan-Heijnen 2006; Gomez et al. 1998). Moreover, patients who have experienced an initial neutropenic event are at increased risk for additional neutropenic events. Neutropenic or infectious events often generate a response from treating clinicians in the management of the subsequent cycles with implementing one or more of the following options: (1) addition of prophylactic use of colony stimulating factors (CSFs); (2) prophylactic use of antibiotics; (3) reduction of chemotherapy dose; (4) delay in the initiation of the next treatment cycle; (5) interruption and/or cessation of the planned regimen. Execution of any of these measures may decrease the risk of neutropenic complications observed in the subsequent cycles (Timmer-Bonte and Tjan-Heijnen 2006). However, in a curative setting such as ESBC, a decrease in chemotherapy dose intensity may result in compromised survival rates (Bonadonna et al. 1995; Budman et al. 1998).

Patterns of utilization of supportive measures implemented in efforts to reduce neutropenic events during the course of chemotherapy within routine community clinical practice are largely unknown. The general population receiving chemotherapy includes patients with comorbidities, frail or elderly, and may differ from patients with cancer treated in randomized control trials (RCTs) who are often healthier and highly preselected (Murthy et al. 2004). The main aim of this study was to assess timing of neutropenic events in relation to clinical interventions such as modification of chemotherapy dose or schedule or addition of supportive care in patients receiving chemotherapy treatment in oncology practice within a community setting. The data from a prospective observational registry study were utilized (Crawford et al. 2008) and the report evaluating all patients with solid tumors or lymphoma was published earlier (Culakova et al. 2014). This analysis is focused on patients with stage I–III breast cancer.

## Methods

### Study description

A prospective observational, multi-center cohort registry study comprised of adult ambulatory patients with solid tumors or lymphoma receiving chemotherapy was conducted between 2002 and 2006. The primary goals of the study were to address questions about the frequency and severity of treatment-related complications and the use of supportive care in cancer patients treated in community-based practices. There was no treatment intervention mandated by the study and choice of the chemotherapy regimen and adjunctive supportive care was at the discretion of the treating oncologist. Patient eligibility was generally not restrictive in order to enroll typical patients cared for in community oncology practices, including those who would not be eligible for most clinical trials because of advanced age or preexisting comorbidities. The study protocol was approved by the University of Rochester Research Subjects’ Review Board (Rochester, NY, USA). Data analyses, interpretation, and reporting were performed at the Study Coordinating Center independent of the funding agency.

### Patient population

Participating patients had to be at least 18 years old with a diagnosis of ESBC starting a new myelosuppressive chemotherapy regimen and anticipated to receive at least four cycles. There was no upper age limit for participation and no restrictions based on the comorbid conditions or performance status. The patients had to be willing to return for scheduled nadir visits and to sign informed consent. In order to lessen the chance of selection bias, sites were required to enroll consecutive eligible patients and maintain a log of all patients who were offered participation in the study. Lactating females, patients treated with continuous chemotherapy, with active infection, receiving concurrent cytotoxic therapy for non-cancer condition, or participating in a double blind clinical trial were excluded. The analysis presented here is based on patients with available toxicity data for at least the first cycle of chemotherapy.

### Study variables

Demographic information, patient and disease characteristics, comorbid conditions, performance status and data concerning the planned chemotherapy treatment were gathered at baseline prior to chemotherapy initiation. Laboratory data, chemotherapy information, and concomitant medications were collected at the start of each cycle for the first four cycles of chemotherapy. Treatment modifications including dose reductions and delays as well as supportive care interventions such as use of antibiotics and CSFs were captured for each cycle. Additionally, laboratory data were collected at the time of expected lowest neutrophil counts (nadir), and during midcycle visits. Adverse events and chemotherapy-associated toxicities, including fever and infection, were most often recorded at the start of the subsequent cycle of chemotherapy. Therefore, some data related to adverse events and toxicities for cycle 4 may be incomplete. Infection was determined based on the report of the treating physician. Similarly, fever was defined as reported by a clinician or the record of a temperature >38.1°C.

Based on type of drugs, dosing and schedule recorded at the baseline, chemotherapy regimens were matched with recognized standard regimens recommended for treatment of ESBC by American Society of Clinical Oncology or National Comprehensive Cancer Network guidelines in order to determine RDI. The planned, current cycle and overall actual RDI were calculated based on the doses and schedule planned at the initiation, cycle related, and given over the course of four cycles, respectively (Culakova et al. 2014).

### Definition of outcomes

Outcomes of interest for this analysis included occurrence of FN, occurrence of severe or febrile neutropenia (SN/FN), reductions in RDI, utilization of CSF and/or antibiotics as primary prophylaxis or overall. SN was defined as absolute neutrophil count (ANC) <500/mm^3^ and FN as ANC <1,000/mm^3^ with reported fever or infection in the same chemotherapy cycle. Primary CSF prophylaxis was defined as CSF use planned at the beginning of the first cycle or prior to a neutropenic event within the first cycle. For subsequent cycles, patients were considered to be treated prophylactically with CSF if it was reported before or at the baseline of the cycle.

### Statistical methods

The goal of this prospective cohort study was to describe the frequency and time course of treatment-related complications and patterns of clinical management including supportive care and was, therefore, primarily descriptive in nature. Clinically relevant variables were summarized using standard descriptive statistics utilizing proportions for categorical variables, and measures of central tendency and variability summarized as mean, median, and/or standard error (SE) for continuous variables. Neutropenic events were assessed as proportions of patients with an event and were measured by cycle as well as cumulatively across cycles. Similarly, proportions of patients receiving supportive care were reported. To evaluate the timing of neutropenic events and clinical interventions aimed at preventing them, hazard rates were estimated using actuarial methods. Statistical analysis was conducted using SAS version 9.3 (SAS Institute Inc.; Cary, NC, USA).

## Results

### Study participants

This prospective cohort study enrolled 4,458 patients with cancer, including 1,224 with ESBC, from 115 randomly selected practice sites located through the entire US (37 in Central US, 22 in Northeast, 34 in South, and 22 in West Coast). The analysis presented here is based on 1,202 patients (1,996 women and 6 men) with ESBC where 22 patients with no toxicity data were excluded. Median and mean age were 53 years (range 26–85, SE = 0.3), nearly 60% of women were post-menopausal and two-thirds had hormone receptor-positive disease. The most common comorbidity reported was diabetes (8.0%), while 82.8% of patients had Charlson Comorbidity Index of zero. More than 90% (n = 1,104) of patients received the first line of chemotherapy which included an anthracycline in 942 (85.3%). Patient demographics, clinical, disease and treatment characteristics are summarized in Table 1 for the entire cohort and separately based on hormone receptor status.

**Table 1:** Descriptive characteristics of the entire cohort and stratified by hormonal status

Characteristics	All patients	ER+ or PR+	ER− and PR−
	n (%)	n (%)	n (%)
Total^a^	1,202	767	387
Age			
<50 years	452 (37.6)	281 (36.6)	150 (38.8)
50–64 years	546 (45.4)	357 (46.5)	170 (43.9)
≥65 years	204 (17.0)	129 (16.8)	67 (17.3)
Menopausal status^a^			
Pre-menopausal	486 (40.7)	316 (41.5)	146 (37.8)
Post-menopausal	709 (59.3)	446 (58.5)	240 (62.2)
Race			
White	961 (80.0)	629 (82.0)	297 (76.7)
Black	153 (12.7)	88 (11.5)	58 (15.0)
Other	88 (7.3)	50 (6.5)	32 (8.3)
Baseline body surface area			
≤2 m^2^	930 (77.4)	595 (77.6)	298 (77.0)
>2 m^2^	272 (22.6)	172 (22.4)	89 (23.0)
Body mass index^a^			
<30 kg/m^2^	769 (64.1)	499 (65.3)	238 (61.5)
30 to <35 kg/m^2^	226 (18.8)	136 (17.8)	81 (20.9)
≥35 kg/m^2^	204 (17.0)	129 (16.9)	68 (17.6)
ECOG performance status			
0	937 (78.0)	608 (79.3)	295 (76.2)
1	241 (20.0)	145 (18.9)	84 (21.7)
2–4	24 (2.0)	14 (1.8)	8 (2.1)
Stage^a^			
I	288 (24.1)	188 (24.6)	96 (25.0)
II	678 (56.7)	437 (57.1)	218 (56.8)
III	230 (19.2)	140 (18.3)	70 (18.2)
Medical history			
Prior chemotherapy	98 (8.2)	49 (6.4)	46 (11.9)
Recent surgery	565 (47.0)	354 (46.2)	188 (48.6)
Diabetes	96 (8.0)	55 (7.2)	36 (9.3)
CHF or MI	24 (2.0)	16 (2.1)	5 (1.3)
Lung disease	30 (2.5)	18 (2.3)	11 (2.8)
History of anemia	85 (7.1)	57 (7.4)	23 (5.9)
Number of comorbidities			
0	1,027 (85.4)	659 (85.9)	329 (85.0)
1	153 (12.7)	97 (12.6)	49 (12.7)
≥2	22 (1.8)	11 (1.4)	9 (2.3)
Chemotherapy treatment			
Standard AC or EC	527 (43.8)	334 (43.5)	170 (43.9)
Dose dense AC or EC	137 (11.4)	82 (10.7)	51 (13.2)
CMF	123 (10.2)	90 (11.7)	31 (8.0)
CAF or CEF	155 (12.9)	105 (13.7)	47 (12.1)
TAC or TEC	83 (6.9)	54 (7.0)	27 (7.0)
Paclitaxel/docetaxel	68 (5.7)	41 (5.3)	25 (6.5)
AT or ET	42 (3.5)	22 (2.9)	16 (4.1)
Other	67 (5.6)	39 (5.1)	20 (5.2)

*ECOG* Eastern Cooperative Group performance status, *ER* estrogen receptor, *PR* progesterone receptor, *MI* myocardial infarction, *CHF* congestive heart failure, *A* doxorubicin, *C* cyclophosphamide, *E* epirubicin, *F* 5-flourouracil, *M* methotrexate, *T* docetaxel.

^a^For some variables sample size is smaller due to missing data, including 48 patients with unknown hormonal status.

### Neutropenic and infectious events

**Fig. 1:**
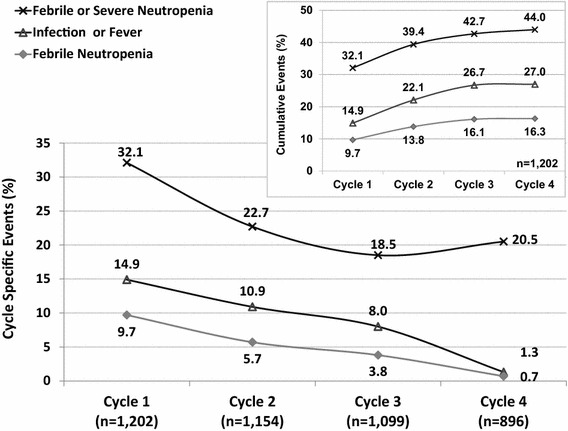
Neutropenic and infectious events during first 4 cycles. Cycle specific events and cumulative events are presented.

Neutropenic and infectious episodes had the highest occurrence during the first cycle of chemotherapy. FN events decreased from 9.7% in cycle 1 to 5.7% and 3.8% in cycles 2 and 3, respectively. Among 196 (16.3%) patients who had a FN event during the treatment, almost 60% (n = 116) experienced the initial event during cycle 1. Overall, 325 (27.0%) patients experienced fever or infection during the observation period and for a majority (179 out of 325) the initial events occurred in cycle 1. Lower rates of infectious events (10.9 and 8.0%) were observed in cycles 2 and 3, respectively, compared to 14.9% in cycle 1. The same patterns were observed for composite events of SN/FN where the incidence fell from 32.1% in cycle 1 to approximately 20% in later cycles. Overall, 529 (44.0%) patients had SN/FN events during the four cycles of chemotherapy and nearly three-quarters experienced their initial event in the first cycle (Fig. 1). The decreasing trend of FN events was observed for pre-menopausal (9.9, 4.7, and 3.3% in cycle 1, 2, and 3, respectively) as well as post-menopausal women (9.6, 6.5, and 4.3% in cycle 1, 2, and 3, respectively) and also for patients with hormone receptor-positive (8.9, 5.2, and 4.0% in cycle 1, 2, and 3, respectively) as well as -negative disease (11.4, 6.7, and 3.3% in cycle 1, 2, and 3, respectively). While the overall incidence of FN did not differ significantly for pre- and post-menopausal women (15.4 vs. 17.1%) or by positive vs. negative hormone receptor status (15.3 vs. 18.1%), the majority of patients in all these subgroups experienced their initial FN during the first cycle. Infectious events and SN/FN events had similar pattern.

### Relative dose intensity

For 1,176 (97.8%) patients, it was possible to match the treatment regimen with published standard regimen and to calculate the RDI. While only 13.4% of patients started chemotherapy with RDI <85%, the overall actual RDI <85% was observed in 22.2% and RDI fell below 85% of standard in at least one cycle in 32.5% of patients (Table 2). The planned RDI <85% was more frequent for patients with body surface area (BSA) >2 m^2^ (22.4%) compared to patients with BSA ≤2 m^2^ (10.7%). Approximately 40% of patients with body mass index (BMI) ≥30 kg/m^2^ had RDI reduced below 85% in at least one cycle compared to 28.1% of patients with BMI <30 kg/m^2^. Reduced RDI at the initiation of the treatment as well as subsequent reductions were more common in older patients and patients with a poor performance status. RDI was similar for patients with hormone receptor-positive and -negative disease.

**Table 2:** Patients with reduced relative dose intensity (RDI) (percent of patients with reductions are presented)

	Planned RDI <85% of standard (%)	Overall actual RDI <85% (%)	RDI <85% for 1+ cycle (%)	Planned RDI <90% of standard (%)	Overall actual RDI <90% (%)	RDI <90% for 1+ cycle (%)
All (n = 1,176)^a^	13.4	22.2	32.5	17.0	30.5	40.0
Age						
<50 years (n = 443)	12.4	16.0	26.0	15.3	23.5	33.2
50–64 years (n = 537)	12.5	23.1	34.3	16.0	32.2	40.8
≥65 years (n = 196)	17.9	33.7	42.3	23.5	41.8	53.1
Menopausal status^a^						
Pre-menopausal (n = 479)	10.4	14.4	23.4	12.9	21.7	30.3
Post-menopausal (n = 690)	15.2	27.4	38.6	19.7	36.4	46.4
ECOG performance status						
0 (n = 917)	12.5	20.9	31.2	15.8	28.8	37.7
1 (n = 235)	15.3	26.4	35.7	20.0	36.6	47.2
2–4 (n = 24)	25.0	29.2	50.0	33.3	37.5	54.2
Prior chemotherapy						
No (n = 1,085)	13.1	21.9	32.4	16.7	30.1	39.9
Yes (n = 91)	16.5	25.3	34.1	20.9	35.2	40.7
Baseline body surface area						
≤2 m^2^ (n = 908)	10.7	19.9	30.3	13.9	28.2	38.0
>2 m^2^ (n = 268)	22.4	29.9	39.9	27.6	38.4	46.6
Body mass index^a^						
<30 kg/m^2^ (n = 750)	9.5	17.9	28.1	12.5	25.6	35.3
30 to <35 kg/m^2^ (n = 220)	16.8	30.0	38.6	20.0	36.8	45.0
≥35 kg/m^2^ (n = 203)	23.6	29.6	41.9	30.0	41.9	51.7
Hormone receptors^a^						
ER+ or PR+ (n = 753)	13.9	22.0	32.9	18.1	30.3	41.2
ER− and PR− (n = 378)	11.6	22.2	30.4	14.3	29.9	36.5

*ECOG* Eastern Cooperative Group performance status, *ER* estrogen receptor, *PR* progesterone receptor, *1+ cycle* at least one cycle.

^a^For some variables sample size is smaller due to missing data.

### Supportive care measures

**Fig. 2:**
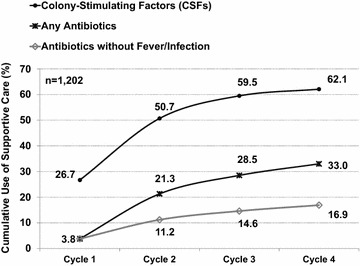
Cumulative use of colony-stimulating factors and antibiotics initiated by the start of a specific cycle.

Approximately one-fourth (26.7%) of patients received primary prophylaxis with CSFs. The use of CSFs nearly doubled in cycle 2, with nearly two-thirds of patients (62.1%) receiving CSFs at some point during the four cycles of chemotherapy. A similar trend was noted for the use of antibiotics. While the use of prophylactic antibiotics in cycle 1 was low (3.8%), overall 16.9% of patients received antibiotics without the evidence of infection or fever (Fig. 2).

### First cycle and subsequent cycles

**Fig. 3:**
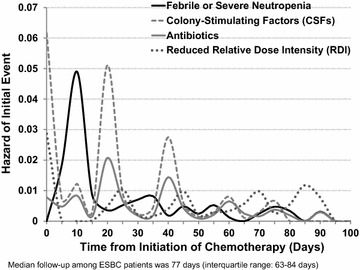
Hazard rates of neutropenic events related to hazard rates of measures implemented to avoid neutropenic complications.

Decreasing rates of FN and infection in later cycles correlated with greater reductions in chemotherapy dose intensity along with increased use of prophylactic CSFs and antibiotics. Figure 3 displays the hazard rates and it compares the timing of the initial neutropenic events to the timing of implementation of the measures used to prevent them.

**Fig. 4:**
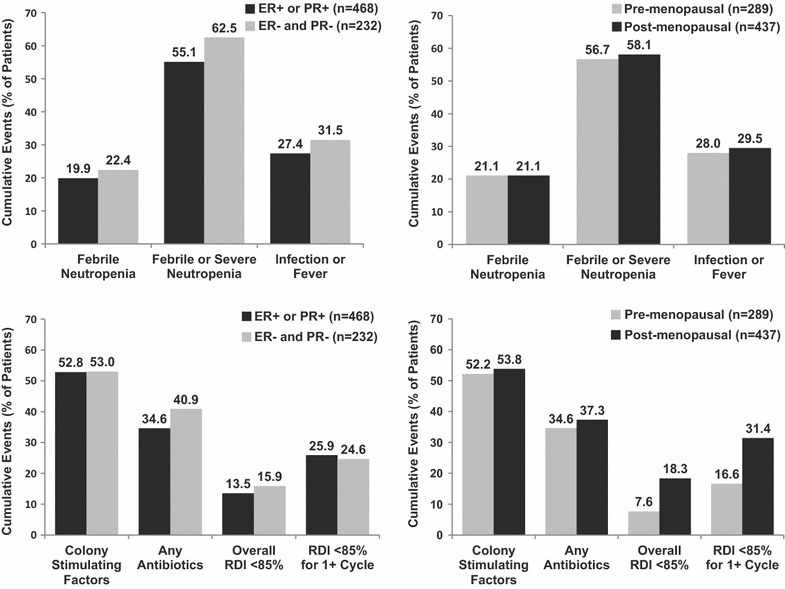
Neutropenic events versus supportive measures in subgroup of patients with planned RDI ≥85% and no primary prophylaxis with colony-stimulating factors. Among 729 patients in this subgroup 29 had unknown hormone receptors status and 3 had unknown menopausal status. *ER* estrogen receptor, *PR* progesterone receptor, *RDI* relative dose intensity, *1+ cycle* at least one cycle.

Reduced dose intensity was associated with fewer FN events. For the group of 157 patients who started treatment with planned RDI <85% of standard, rates of FN events in cycle 1 were 4.5 and 8.9% overall. In contrast, the FN rates were significantly higher (10.6% in cycle 1 and 17.6% during four cycles) for patients with planned RDI ≥85% (n = 1,019). The overall FN rates reached 21.0% in 729 patients who started chemotherapy with planned RDI ≥85% without primary CSF prophylaxis. In this group, the actual overall RDI fell below 85% more frequently in post-menopausal patients than pre-menopausal patients (18.3 vs. 7.6%) (Fig. 4). There was no significant difference in FN rates, use of antibiotics or addition of CSFs by menopausal or hormone receptor status.

### Toxicities and treatment completion

**Fig. 5:**
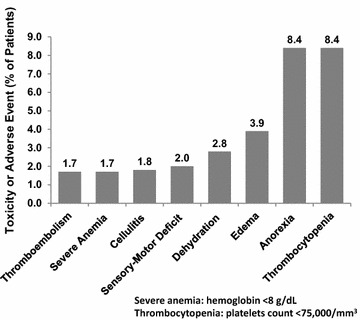
Serious chemotherapy related non-myeloid toxicities and adverse events.

In addition to neutropenic complications, the most common reported adverse events were gastrointestinal (nausea 54.4%, vomiting 24.3%, diarrhea 14.1%), fatigue (50.2%), myalgia (15.1%), and pain (14.8%). Treatment-related anemia (hemoglobin <10 g/dL) was observed in one-quarter of the patients (25.4%), with hemoglobin levels <8 g/dL in 21 individuals (1.7%) (Fig. 5). Of 1,202 ESBC patients, 1,064 (88.5%) completed at least four cycles of chemotherapy and, of those, 168 had missing toxicity data related to the nadir of cycle 4. Reasons for not completing four cycles included treatment toxicity (n = 26), patient-requested withdrawal (n = 15), disease progression (n = 15), other medical reasons (n = 12), and administrative/protocol-related progression or loss to follow-up (n = 67). Administrative reasons for dropping out of the study early were related to the study protocol including requirements for nadir laboratory tests and did not necessarily result in termination of chemotherapy. Three patients died within the first four cycles of chemotherapy with reported causes of death being cardiac arrest, pulmonary emboli, and sepsis.

## Discussion

In this prospective cohort study of ESBC patients receiving conventional chemotherapy in a community practice setting, the risk of the initial neutropenic complications was greatest during the first cycle of chemotherapy. This is consistent with reports from other studies (Vogel et al. 2005; Haim et al. 2005; Chan et al. 2011; Martin et al. 2006). It is in line with earlier reports utilizing data from this prospective study, and it applies across tumors types (Crawford et al. 2008; Culakova et al. 2014).

At the initiation of treatment, most patients received the full dose of chemotherapy, often with no primary prophylaxis with CSFs or antibiotics. The decrease in neutropenic events during subsequent cycles was likely the result of clinical interventions such as reductions in chemotherapy dose intensity and/or the additional use of supportive care measures prompted by neutropenic events in cycle 1. A recent trial by Aarts et al. tested the hypothesis that in order to decrease treatment cost, the use of granulocyte colony-stimulating factor (G-CSF) may be limited to the initial cycles (Aarts et al. 2013). Breast cancer patients with estimated FN risk above 20% were randomized to G-CSF prophylaxis with pegfilgrastim given only in cycle 1 and 2 (experimental arm) versus pegfilgrastim in all cycles. In contrast to the expected results, in the experimental arm, FN rates climbed after G-CSF was discontinued to the maximum of 24% in the third cycle, and reached overall risk of 36%, compared to 10% rates in the standard arm with continued prophylaxis.

Hematologic toxicities as well as chemotherapy dose intensity are often underreported in the published reports of clinical trials (Lyman 2009; Dale et al. 2003; Truong et al. 2015). A recent systematic review by Younis et al. (2012) studied FN rates outside of RCTs for two commonly used regimens: (1) docetaxel 75 mg/m^2^, cyclophosphamide 600 mg/m^2^ every 21 days for four cycles (TC) and (2) 5-fluorouracil 500 mg/m^2^, epirubicin 100 mg/m^2^, and cyclophosphamide 500 mg/m^2^ on day 1 every 21 days for three cycles followed by docetaxel 100 mg/m^2^ day 1 every 21 days for three cycles (FEC-D). The average FN rate was 17% (range 7–33%) for 13 studies with TC regimen reaching 29% when prophylactic G-CSF was not given, compared to 5% reported in the trial (Younis et al. 2012; Jones et al. 2006). Similarly, for nine retrospective studies of FEC-D treatment, the FN rate was 24% (18–35%) reaching 31% without G-CSF prophylaxis compared to 11.2% reported in the PACS-01 trial (Younis et al. 2012; Roche et al. 2006). In the study reported here, 16% of patients developed FN over four cycles. While one-fourth of patients received prophylactic CSFs initially, clinical events resulted in the use of CSFs in nearly two-thirds of patients over the period of observation. In patients initiating treatment with full dose chemotherapy and without G-CSF support, the rate of FN exceeded 20% and approximately one-half received G-CSF after cycle 1. Of note, there were no meaningful differences in G-CSF use or in FN events based on menopausal or hormone receptor status in this group.

The safety and efficacy of primary prophylaxis with G-CSF in patients with solid tumors including breast cancer receiving chemotherapy was established in RCTs (Vogel et al. 2005; Timmer-Bonte et al. 2005; Lyman et al. 2015a). A meta-analysis of RCTs of chemotherapy with or without prophylactic G-CSF confirmed significant reductions in FN, early mortality, and infection-related mortality (Kuderer et al. 2007). In addition, a systematic review of RCTs in patients with breast cancer demonstrated significant reductions in FN, early mortality, risk of hospitalization, and use of intravenous antibiotics (Renner et al. 2012). Another meta-analysis reported improved overall survival with more intense breast cancer regimens supported by G-CSF (Lyman et al. 2013b). While routine prophylactic CSFs are recommended in patients at 20% or greater risk of FN, the use of prophylactic antibiotics is discouraged due to concerns over rising rates of antimicrobial resistance (Aapro et al. 2011; Crawford et al. 2013; Smith et al. 2006). Current guidelines oppose antimicrobial prophylaxis unless prolonged severe neutropenia is expected (Flowers et al. 2013). In the study reported here, 4% of patients received prophylactic antibiotics in the absence of fever or infection in cycle 1 increasing to 17% over the four cycles observed.

Although neutropenia commonly results from cytotoxic chemotherapy and lowering chemotherapy dose intensity may reduce the risk of FN, reductions in chemotherapy dose intensity may compromise long term outcome and shorten overall survival. RCTs as well as observational studies have demonstrated the importance of maintaining chemotherapy dose intensity in the curative setting of ESBC (Bonadonna et al. 1995; Budman et al. 1998; Chirivella et al. 2009; Perez-Fidalgo et al. 2014). In the study reported here, one-fifth of patients received overall RDI below 85% while 13% started the treatment with planned RDI <85%. Of note, reduced RDI was more common for older patients, patients with poor performance status, and obese patients. Planned dose reductions were twice as common with BSA >2 m^2^ compared to those with BSA ≤2 m^2^. Guidelines on appropriate chemotherapy dosing of obese adult patients with cancer recommend dosing based on calculations using actual body weight for most cytotoxic agents (Griggs et al. 2012). Routine dose reductions, such as capping of the doses at 2 m^2^, are discouraged. Most published data indicate no increase in treatment-related toxicities in obese patients with cancer receiving full dosing estimated by using actual weight (Griggs et al. 2012; Carroll et al. 2012). In addition, clinicians are encouraged to use the same rules when responding to the toxicities in non-obese and obese patients (Griggs et al. 2012).

It is important to note limitations to the current study. The population of this prospective observational study received chemotherapy between 2002 and 2006, and may not be representative of chemotherapy regimens and supportive care introduced subsequent to that period. Importantly, toxicity data including fever, infection, and FN for cycle 4 are likely underreported with nearly 20% of patients completing all four cycles missing toxicity data for the last cycle. Also, patients who discontinued chemotherapy prematurely have incomplete data and represent a potentially more vulnerable group with higher risk of adverse events. Additionally, only data during the first four cycles were captured and long term follow-up is not available. Nevertheless, this prospective cohort is the largest prospective study of cancer patients capturing detailed chemotherapy treatment and its complications in the community setting in US oncology practices.

## Conclusions

In summary, while the risk of neutropenic complications is greatest in the initial cycle, the cumulative risk of neutropenic events remains high in patients receiving full dose chemotherapy without prophylactic measures. The apparent reduction in the risk of neutropenic complications in subsequent cycles appears to be associated with efforts to reduce risk through chemotherapy dose reductions and treatment delays or the secondary use of prophylactic measures including CSFs, antibiotics or both. While other patient-, disease-, and treatment-related factors may also influence toxicity patterns, they likely played less of a role in the observed events. Moreover, most ESBC patients are treated with a curative intent and delivery of full-dose chemotherapy is important to sustain favorable long term outcome. Nevertheless, the influence of patient and provider decisions, as well as institutional and payer policies on the observed rates of treatment-related toxicities require additional research (Lyman et al. 2015b). Further, the potential impact of educational efforts, practice guidelines or pathways as well as direct and out of pocket healthcare costs on treatment and supportive care strategies as well as clinical and economic outcomes require further investigation.
